# Supplementary material for: On the limitations in assessing stability of oxygen evolution catalysts using aqueous model electrochemical cells
Source: Nat Commun. 2021 Apr 13;12:2231. doi: 10.1038/s41467-021-22296-9 (PMC8044118; doi:10.1038/s41467-021-22296-9)
Supplement: Supplementary file 1 — Supplementary Information [file 41467_2021_22296_MOESM1_ESM.pdf]

Supplementary information

On the limitations in assessing the stability of oxygen evolution catalysts using aqueous model electrochemical cells

Julius Knöppel[a,b,*], Maximilian Möckl[c], Daniel Escalera-López[a], Kevin Stojanowski[a,b], Markus Bierling[a,b], Thomas Böhm[a,b], Simon Thiele[a,b], Matthias Rzepka[c] and Serhiy Cherevko[a,*]

[a] Forschungszentrum Jülich GmbH, Helmholtz Institute Erlangen-Nürnberg for Renewable Energy (IEK-11), Forschungszentrum Jülich, Egerlandstr. 3, 91058 Erlangen, Germany

[b] Department of Chemical and Biological Engineering, Friedrich-Alexander-Universität Erlangen-Nürnberg, Egerlandstr. 3, 91058 Erlangen, Germany

[c] ZAE Bayern, Electrochemical Energy Storage, Walther-Meißner-Str. 6, 85748, Garching, Germany

j.knoeppel@fz-juelich.de;

s.cherevko@fz-juelich.de

Supplementary Note 1. Precipitation free MEA cell setup

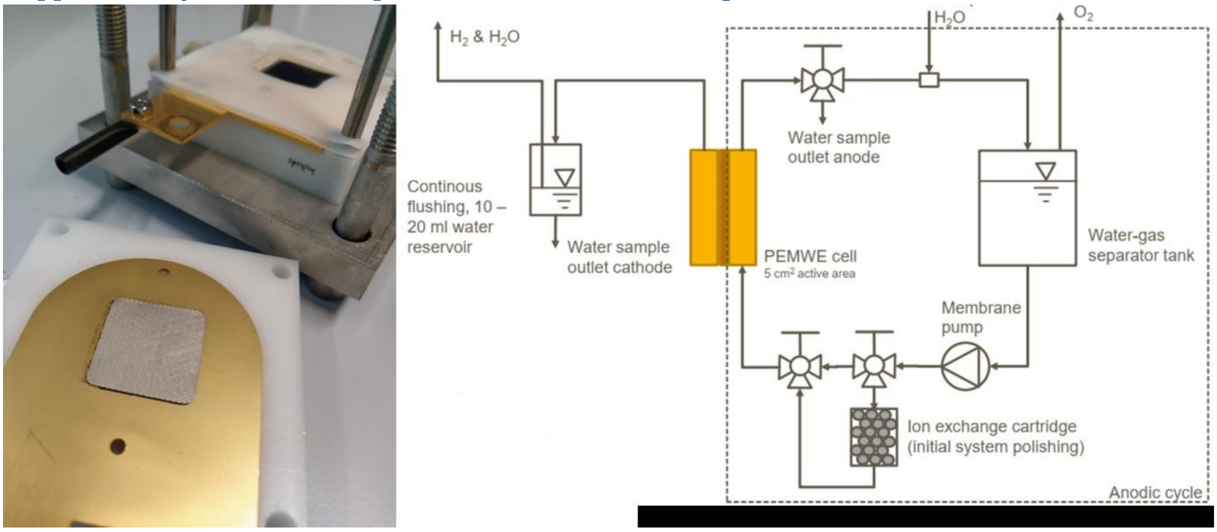

Figure 1 a) Picture of the precipitation free MEA cell setup. The titanium flow fields are gold plated, the anode side porous transport layer is a platinized expanded titanium sheet. The cell body is made from of a PTFE block. b) Flow scheme of the precipitation free MEA cell setup. The setup is polished by shorting cell inlet and outlet and cycling the DI water through an ion exchanger before connecting the cell. Samples are taken from the anode water cycle and the cathode outlet respectively.

Supplementary Note 2. Calculation of dissolution rates and mass losses in MEA and H-cell

Water balance calculations MEA

Experiments in MEA are carried out with refilling the volume taken as samples. Hence, mass loss calculations and dissolution rates have to be calculated.

The water balance calculations for dissolution rates and totally dissolved iridium in the MEA cell setup were carried out as follows.

V_0 Initial water volume in the anode cycle

V_S Sample volume

$\left(\frac{dV_A}{dt}\right)_C$ Volume loss from anode to cathode through electroosmotic drag

$\left(\frac{dV_A}{dt}\right)_R$ Volume loss from anode side through consumption in the reaction

$c_m(t)_A$ Concentration measured at time t in the anode water cycle

$c_m(t)_C$ Concentration measured at time t at the cathode outlet

$c_c(t)_A$ Calculated concentration on the Anode side taking sample tapping and replacement into account.

With continuous refill to keep the water level in the anode cycle constant the mass loss at time t_n there the nth sample is taken is given by

$$m(t_n)_A = c_m(t_n)_A V_0 + c_c(t_{n-1})_A V_S$$

If no constant refill is used and the water level continuously drops by electroosmotic drag and the reaction, the mass loss through the anode side is calculated with

$$m(t_n)_A = c_m(t_n)_A \left(V_0 - \left[\left(\frac{dV}{dt}\right)_C + \left(\frac{dV}{dt}\right)_R \right] (t_n) \right) + c_c(t_{n-1})_A V_S$$

The iridium mass loss through the cathode side is calculated as follows.

$m(t_n)_C = \left(\frac{dV}{dt}\right)_C (t_n - t_{n-1})\, c_m(t_n)_C$

Water balance calculations H-cell

In the H-cell the water balance and mass loss calculations are performed as follows

The current in H-cell is 0.56×10^{-3} A

The mass loss through evolved O_2 in the reaction in 48 hours is

$$m(\mathrm{O_2}) = \frac{MIt}{zF} = 0.008\ \mathrm{g}$$

Hence, the mass loss through the reaction is negligible.

With the variables

V_0 Initial water volume on each side

V_S Sample volume

$c_m(t)$ Concentration measured at time in a respective compartment

$c_c(t)$ Concentration calculated for time t in the respective compartment

In an experiment with refill the iridium mass loss at the anode side the nth data point is given by

$$m(t_n) = c_m(t_n)V_0 + c_c(t_{n-1})_A V_S$$

Supplementary Note 3. Electrochemical and dissolution Data in MEA

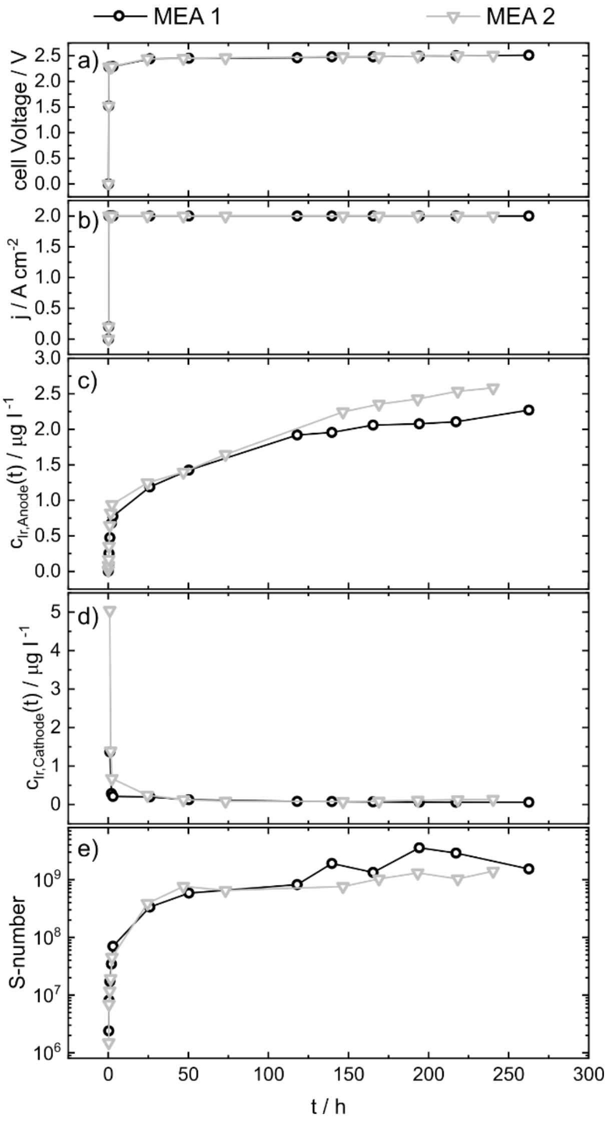

Figure 2 **Electrochemical and dissolution data of the precipitation free MEA. a)** non-iR drop corrected cell potential. **b)** applied current density. **c)** Iridium concentration in the anode water cycle. **d)** Iridium concentration on the cathode water outlet. **e)** S-numbers. The method for calculating dissolution rates, and therefore S-numbers, is shown in supplementary note 5.

Supplementary Note 4. Full SFC Protocol

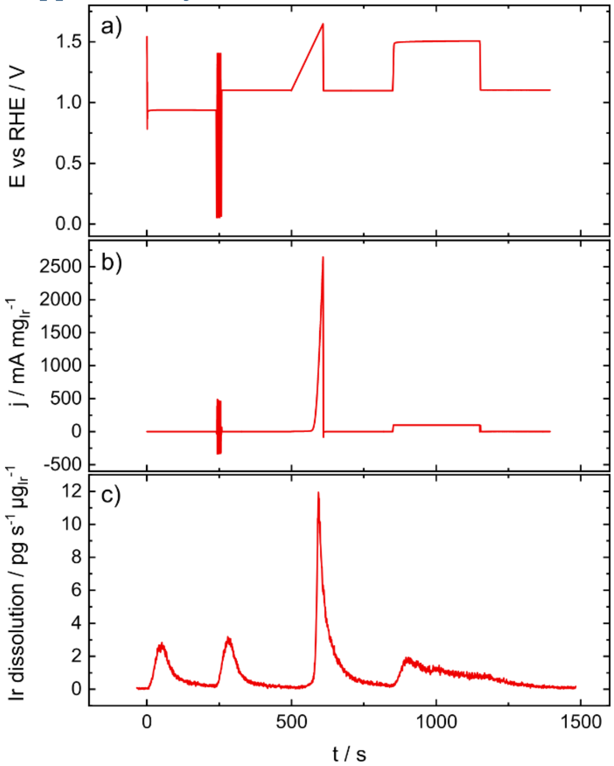

Figure 3 **Full protocol used for all SFC measurements in this study. a)** applied potential **b)** resulting current **c)** resulting dissolution.

The protocol used for all SFC measurements is composed of the following steps:

1. Contact during a 240 s hold at open circuit potential (OCP)
2. 3 fast cleaning CVs from 0.05 V_{RHE} to 1.4 V_{RHE} at a scan rate of 500 mV s^{-1}
3. Hold at 1.1 V_{RHE} for 240 s
4. Ramp from 1.1 V_{RHE} to 1.65 V_{RHE}. Scan rate: 5 mV s^{-1}
5. Hold at 1.1 V_{RHE} for 240 s
6. Galvanostatic hold at 100 mA mg_{Ir}^{-1} for 300 s
7. Hold at 1.1 V_{RHE} for 240 s

All S-numbers reported in this study for SFC-measurements are calculated from a 30 s time period at the end of the galvanostatic hold. For full description, we refer to the main text.

Variation of loading

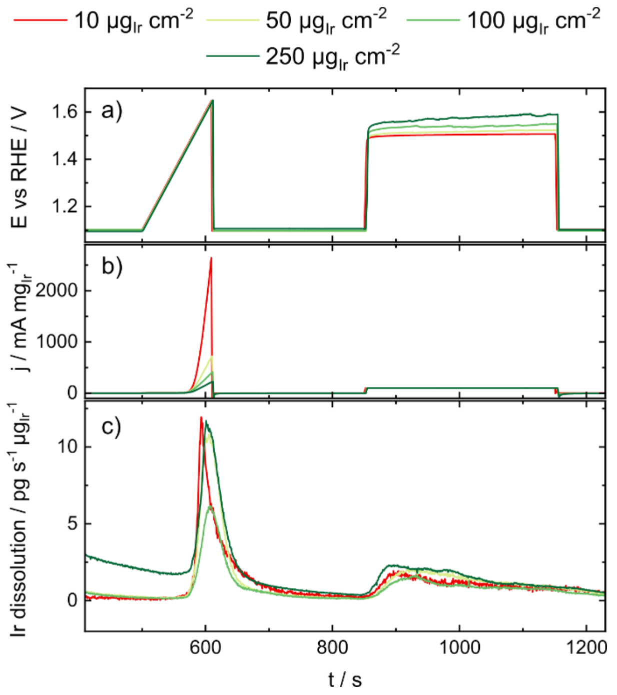

Figure 4 **Non iR-drop corrected electrochemical data and dissolution data with variation of catalyst loading in SFC-ICP-MS. a)** E vs RHE. **b)** gravimetric current density. **c)** resulting loading normalized dissolution. The dissolution curves at the potential step are virtually equivalent.

Variation of flow rate

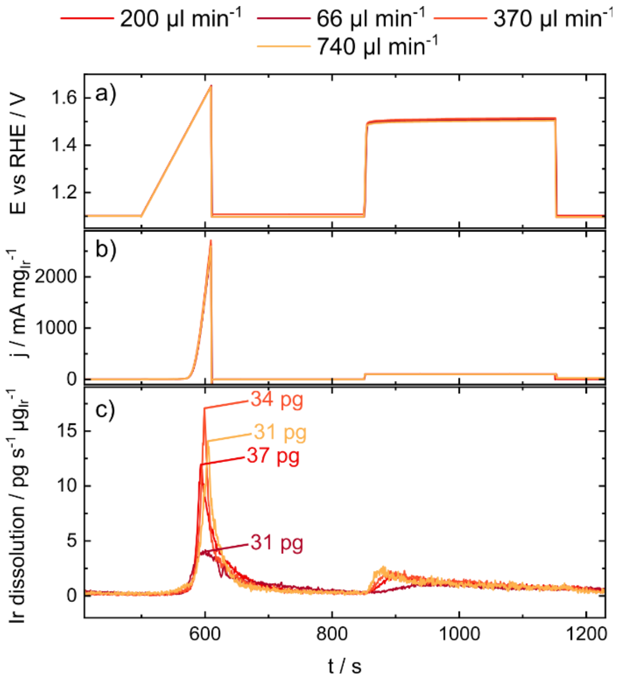

Figure 5 **Non iR-drop corrected electrochemical data and dissolution data with variation of electrolyte flow rate in SFC-ICP-MS a)** E vs RHE. **b)** gravimetric current density**. c)** resulting loading normalized dissolution. The dissolution curves are different in shape due to different tailing properties at varied flow rates [1], but the dissolved amount of iridium is equivalent.

As mass transport properties of dissolved catalyst species from the catalyst surface to the ICP-MS are changing with the flow rate of electrolyte through the cell, a different tailing behaviour is expected at different flow rates [1]. Hence, the dissolution profiles deviate especially at the beginning and the end of the current step. However, after reaching a quasi-steady state towards the end of the applied current pulse, the Ir dissolution rate for all four flow rates is virtually equivalent, along with the calculated S-numbers.

Measurements with electrochemically dissolved iridium in the electrolyte

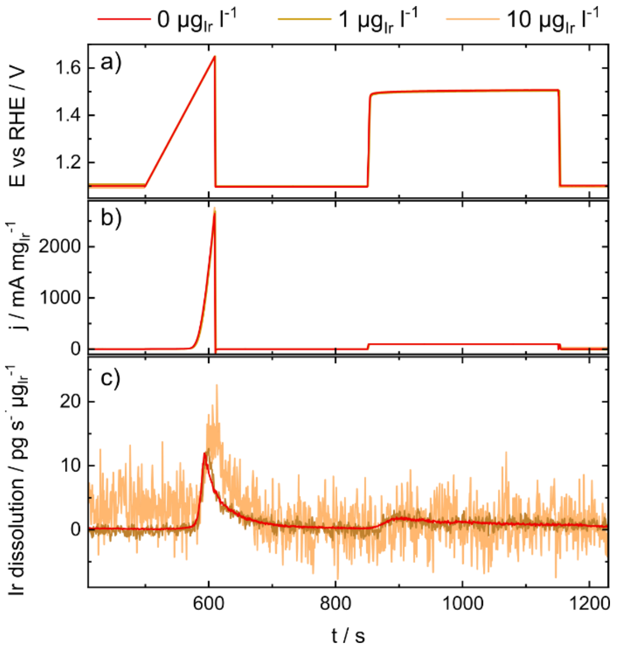

Figure 6 **Non iR-drop corrected electrochemical data and dissolution data with varied amounts of electrochemically dissolved iridium in the electrolyte in SFC-ICP-MS. a)** E vs RHE. **b)** gravimetric current density. **c)** resulting normalized dissolution. The dissolution curves with pre-dissolved iridium in the electrolyte, besides a worsened signal-to-noise ratio, are virtually equivalent to the baseline measurement.

Influence of Nafion content in the catalyst layer on dissolution

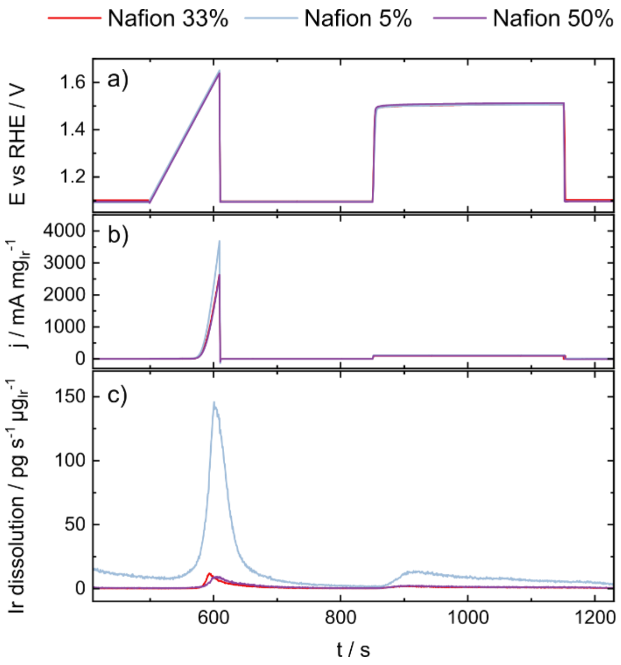

Figure 7 **Non iR-drop corrected electrochemical data and dissolution data with variation of Nafion content in the catalyst layer in SFC-ICP-MS. a)** E vs RHE. **b)** gravimetric current density. **c)** dissolution profiles of catalyst spots with different Nafion content. While catalyst spots with high Nafion content (33%, 50%) exhibit similar dissolution behavior, catalyst spots with low Nafion content dissolve much more at similar current densities.

Influence of pH differences on dissolution

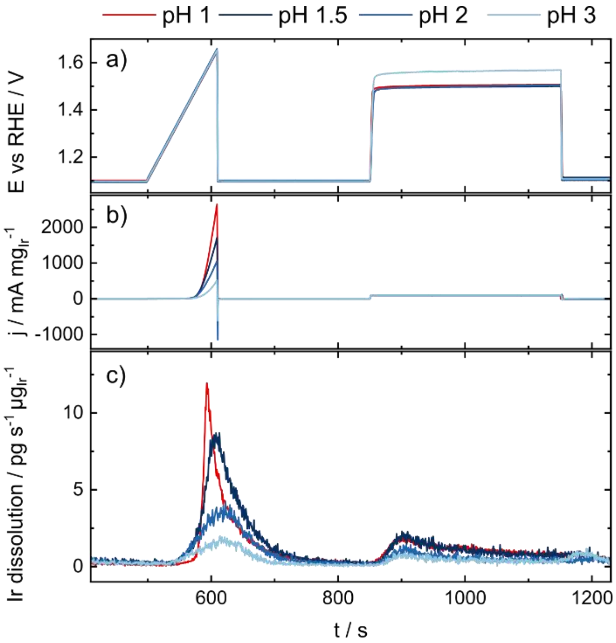

Figure 8 **Non iR-drop corrected electrochemical data and dissolution data with variation of pH in SFC-ICP-MS. a)** E vs RHE. **b)** gravimetric current density. **c)** dissolution data of catalyst spots, measured with electrolyte between pH 1 and pH 3. The dissolution during the galvanostatic step decreases in steadily with increasing pH.

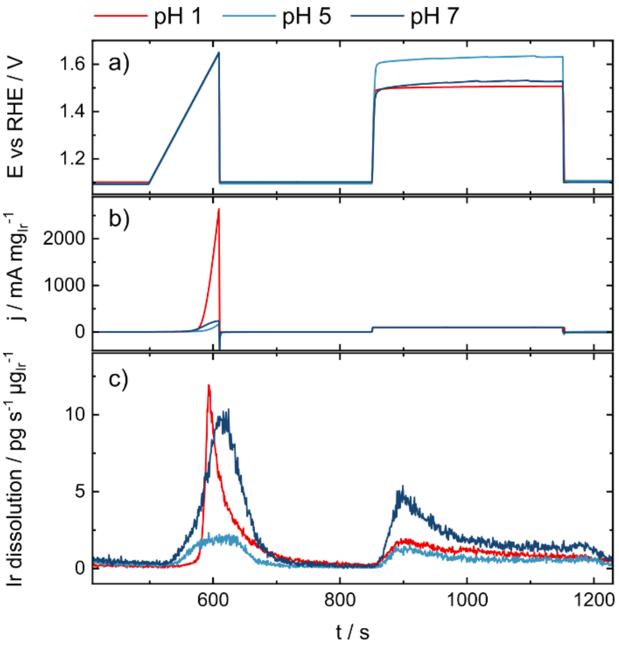

Figure 9 **Non iR-drop corrected electrochemical data and dissolution data with variation of pH in SFC-ICP-MS. a)** E vs RHE. **b)** gravimetric current density. **c)** dissolution data of catalyst spots, measured with electrolyte at pH 1, measured in 0.1 M H_2SO_4 as well as pH 5 and pH 7 measured in 0.05 M phosphate buffer. The results do not show a clear trend. Due to the different electrolytes employed, no clear conclusions can be drawn from the results.

Supplementary Note 6. Additional H-cell Information

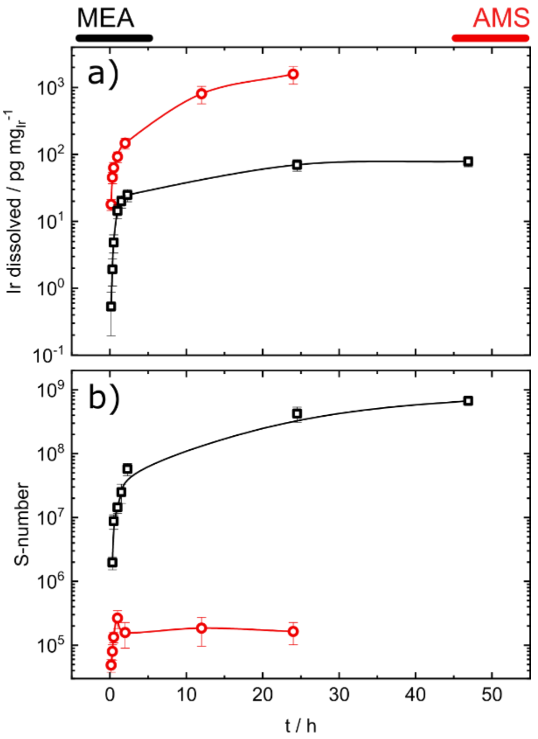

Figure 10 **Long term stability of IrO_x in AES and MEA environment. a)** Loading-normalized total dissolved iridium amount at current densities of 0.2 A mg_{Ir}^{-1} and 2 A mg_{Ir}^{-1} in AES and MEA respectively; b) S-numbers calculated from the amounts of dissolved iridum. Lines are there to guide the eye.

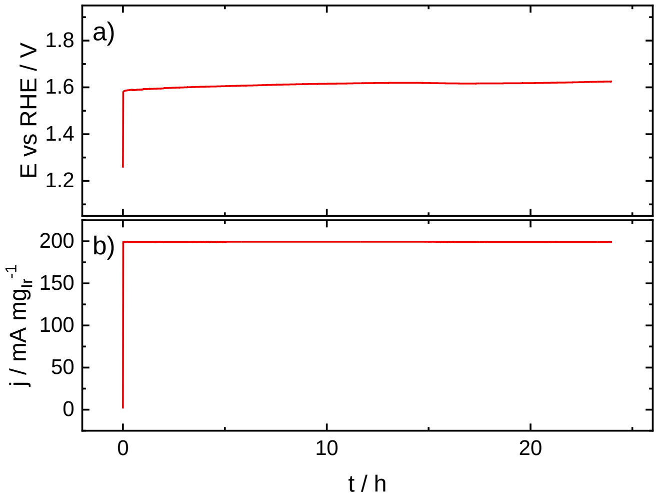

Figure 11 **Exemplary electrochemical H-cell data in a 24 h measurement. a)** Potential versus the reversible hydrogen electrode; **b)** gravimetric current densities.

Supplementary Note 7. STEM analysis of migration processes in MEA.

MEA with DI water in the water cycle, anode side

	Spectrum 1		Spectrum 2		Spectrum 3	
	**Wt%**	**σ**	**Wt%**	**σ**	**Wt%**	**σ**
**Ir**	52.6	1.1	10.9	1.5	1.9	0.7
**C**	25.2	0.9	52.4	1.4	55.0	1.0
**O**	13.5	0.6	3.9	0.7	1.0	0.5
**F**	8.7	0.5	32.8	1.2	42.1	0.9

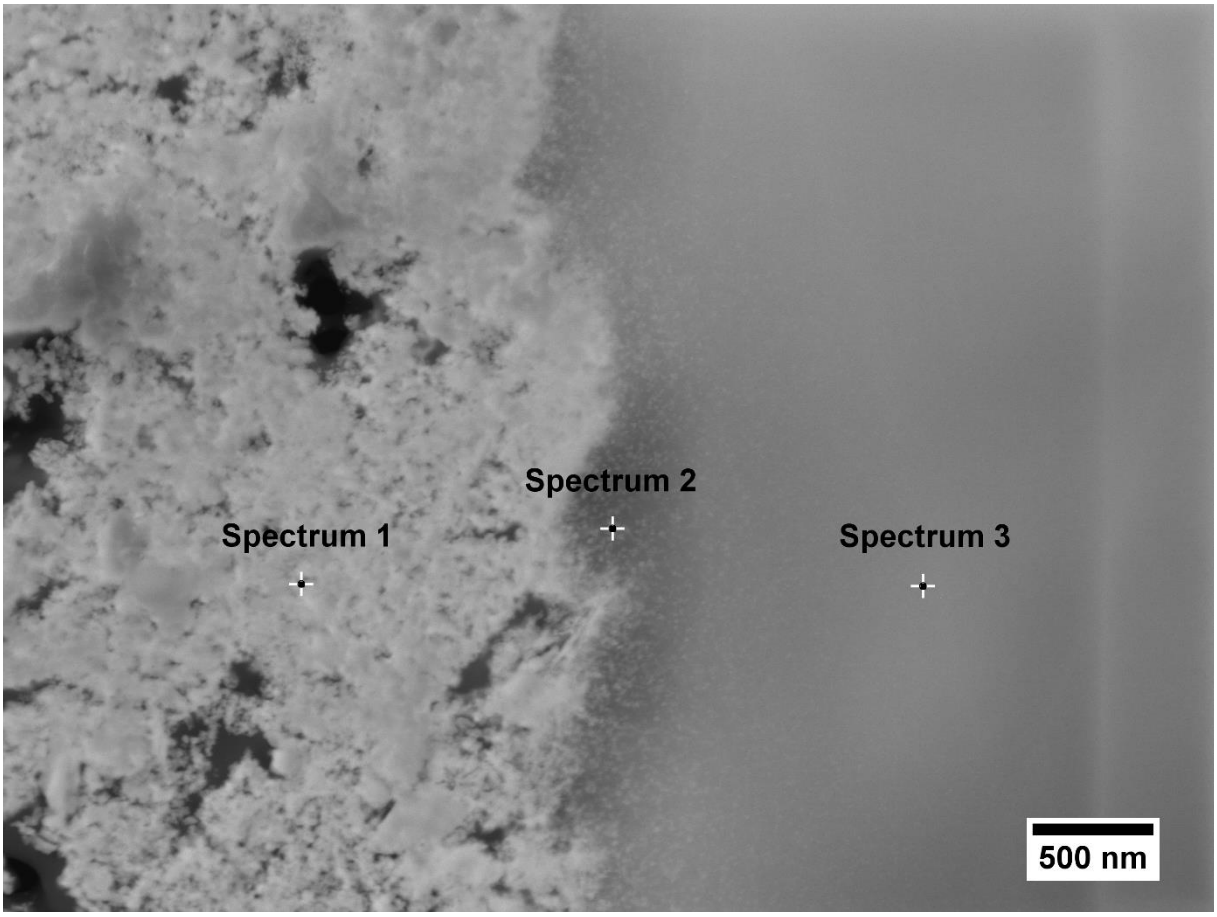

Figure 12 STEM micrograph of the anode catalyst layer of an MEA operated for 48 h at 2 A cm^{-2} with DI water circulating in the anode water cycle.

MEA with DI water in the water cycle, cathode side

	Spectrum 4		Spectrum 5		Spectrum 6	
	**Wt%**	**σ**	**Wt%**	**σ**	**Wt%**	**σ**
**C**	47.1	1.2	51.0	1.0	67.1	0.9
**F**	39.6	1.1	34.4	0.9	13.9	0.6
**O**	12.5	0.9	12.7	0.8	11.5	0.7
**Ir**	0.8	1.5	1.8	1.3	0.0	1.1
**Pt**	0.0	0.4	0.0	0.5	7.5	0.5

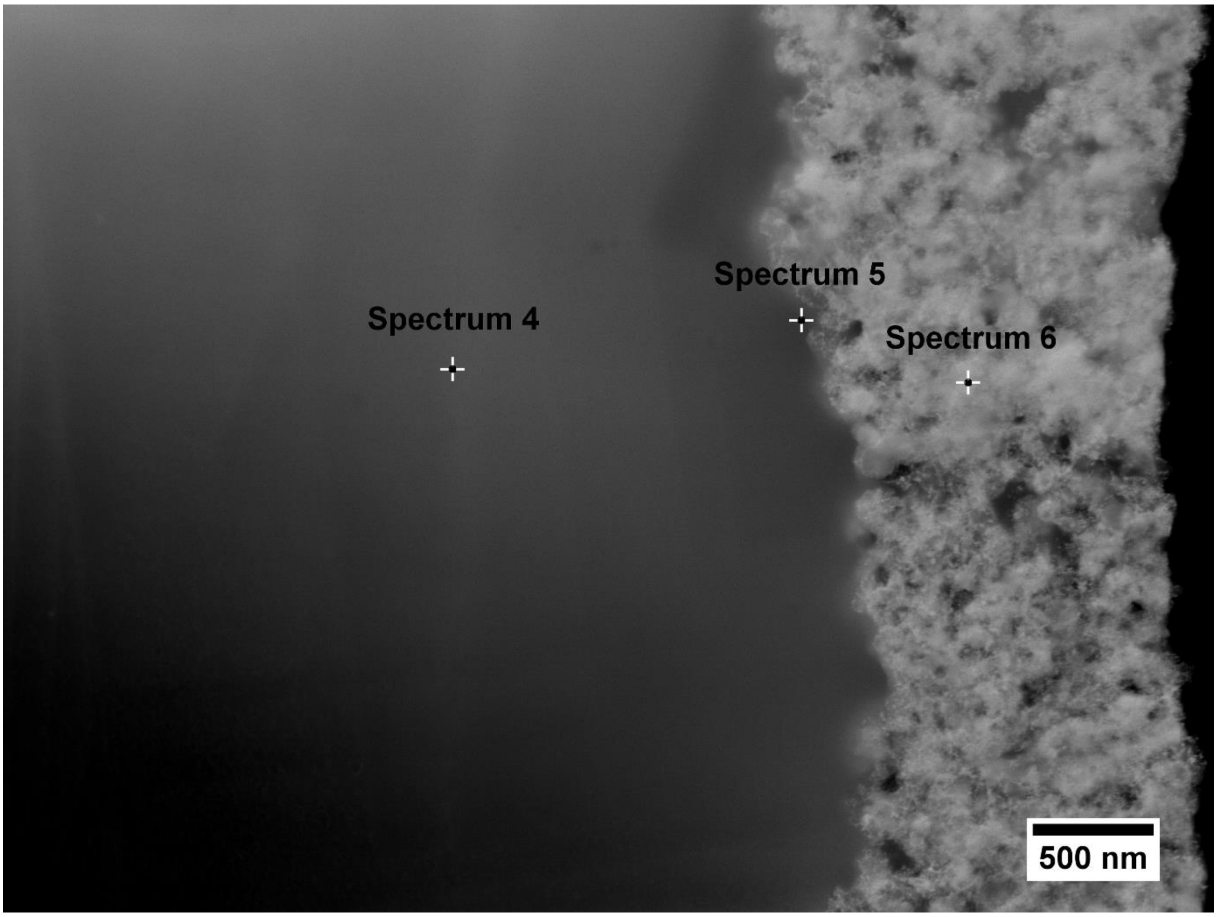

Figure 13 STEM micrograph of the cathode catalyst layer of an MEA operated for 48 h at 2 A cm^{-2} with DI water circulating in the anode water cycle.

MEA operated with diluted acid in the water cycle, anode side

	Spectrum 7		Spectrum 8		Spectrum 9		Spectrum 10	
	**Wt%**	**σ**	**Wt%**	**σ**	**Wt%**	**σ**	**Wt%**	**σ**
**Ir**	41.3	1.9	1.7	1.5	0.0	0.6	0.6	0.8
**C**	31.3	1.5	63.8	2.3	29.9	0.5	60.03	1.3
**O**	16.6	1.1	3.8	1.5	3.3	0.3	2.4	0.7
**Au**	8.1	1.6	0.0	0.9	58.1	0.5	9.0	0.8
**F**	1.6	0.6	30.5	1.9	8.8	0.3	27.6	1.0
**Pt**	0.9	1.3	0.1	0.8	0.0	0.5	0.0	0.1
**Ti**	0.1	0.1	0.0	0.1	0.0	0.1	0.0	0.6

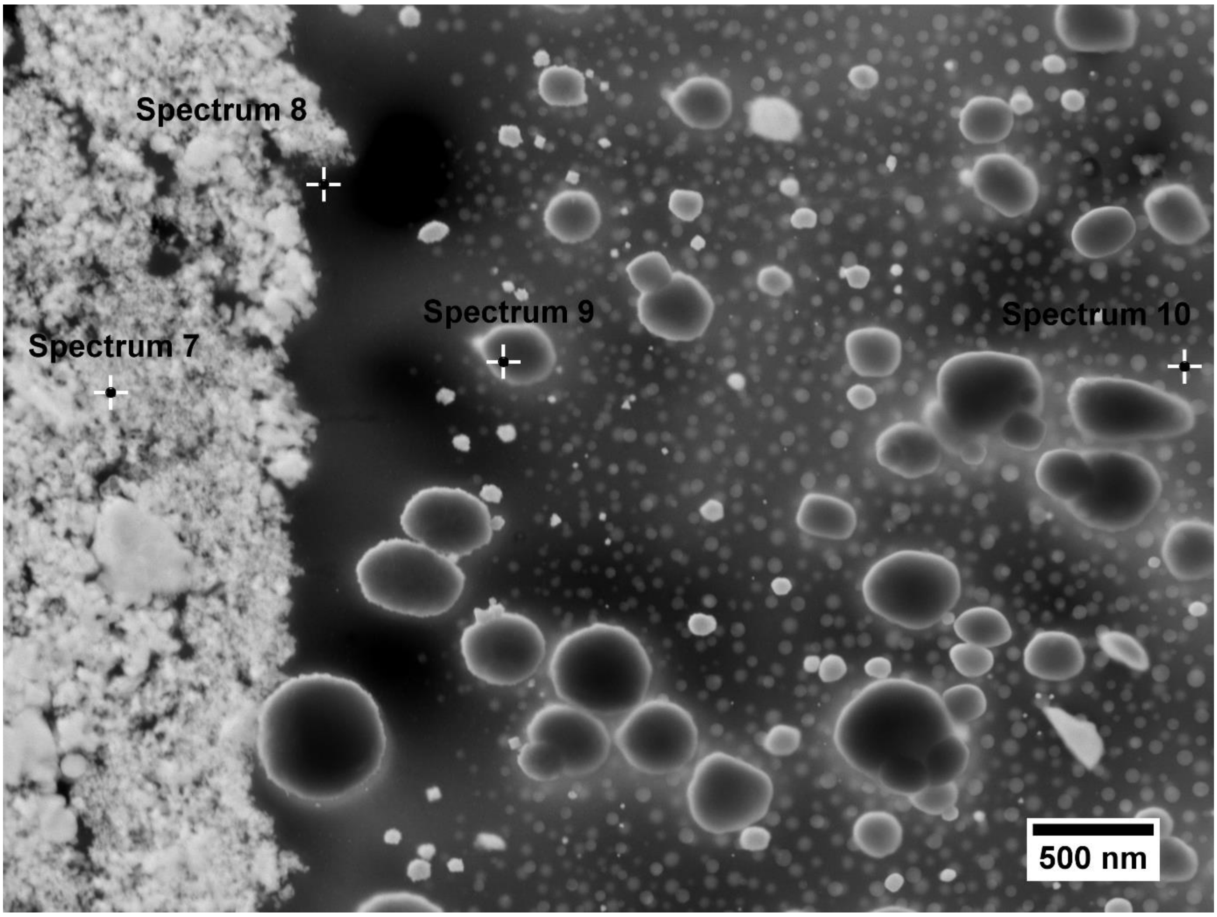

Figure 14 STEM micrograph of the anode catalyst layer of an MEA operated for 48 h at 2 A cm^{-2} with 0.1 M H_2SO_4 circulating in the anode water cycle.

MEA operated with diluted acid in the water cycle, cathode side

	Spectrum 11		Spectrum 12		Spectrum 13		Spectrum 14	
	**Wt%**	**σ**	**Wt%**	**σ**	**Wt%**	**σ**	**Wt%**	**σ**
**C**	54.6	2.0	45.1	1.5	15.3	1.1	55.7	1.2
**F**	26.7	1.4	35.4	1.3	20.4	1.2	8.8	0.6
**Ir**	15.8	2.0	6.7	1.4	7.5	1.2	11.2	1.2
**O**	1.6	1.0	7.3	1.1	39.2	1.3	9.2	0.8
**Au**	1.0	1.0	0.0	0.7	0.0	0.6	0.0	0.9
**Pt**	0.2	0.8	0.1	0.6	0.3	0.5	13.6	0.7
**Ti**	0.2	0.2	5.4	0.3	17.3	0.6	1.4	0.2

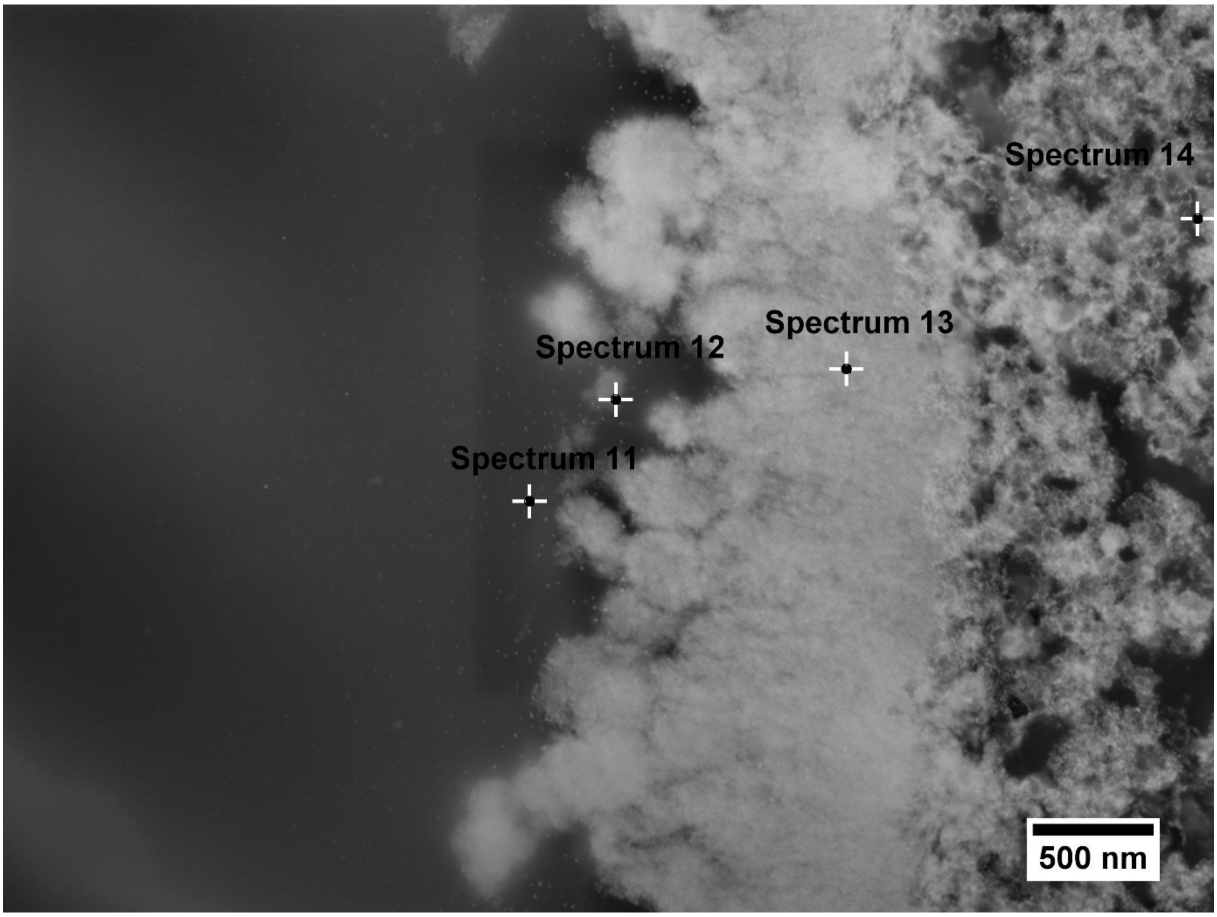

Figure 15 STEM micrograph of the cathode catalyst layer of an MEA operated for 48 h at 2 A cm^{-2} with 0.1 M H_2SO_4 circulating in the anode water cycle.

Supplementary Note 8. Activity changes due to pH shift in aqueous electrolyte and MEA

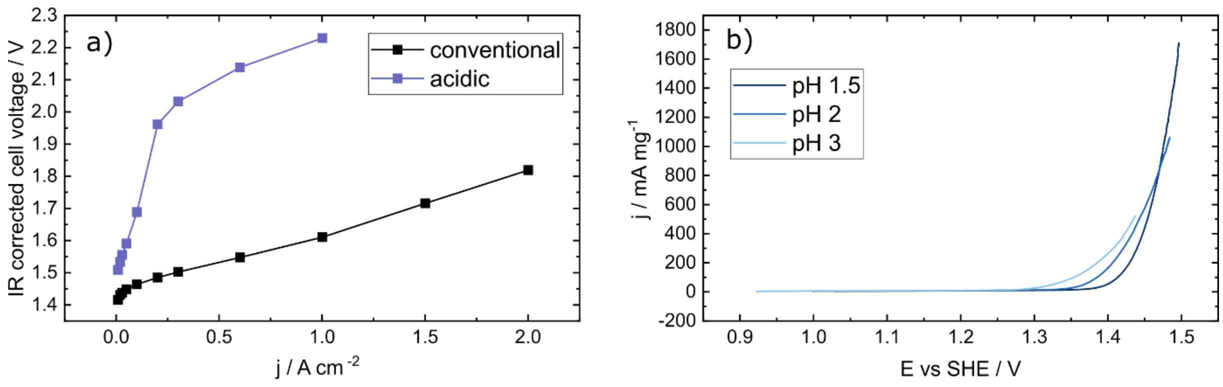

Figure 16 **Changes in activity due to a change in pH a)** iR-drop corrected end of life polarization curves in the precipitation free MEA setup with anode catalyst loadings of 1 μg_{Ir} cm^{-2} with DI water and 0.1 M H_2SO_4 in the anode water cycle **b)** iR-drop corrected ramps at a scan speed of 5 mV s^{-1} of iridium catalyst spots at different pH values.

According to the Nernstian equation, the onset potential of the OER shifts due to changes in pH with 0.059pH towards lower potentials against the standard hydrogen electrode (SHE). As water supply at the cathode side in the MEA system only takes place through electroosmotic flow (proportional to the current density), the proton concentrations as well as the hydrogen pressure are constant at a given current density, the cathode side can be treated as a standard hydrogen electrode. Hence, differences in onset and operating potentials in the MEA system due to reactants of different pH can be accounted to changes due to pH. In figure 12 a) polarization curves of MEAs with DI water and 0.1 M H_2SO_4 as reactants are displayed. Even at low current densities the potential of the acidic curve exceeds the potential of the conventional system by more than 100 mV, indicating a pH shift of 2. At higher potentials the gap increases, indicating either mass transport limitations or even higher pH differences. These results show, that pH in the conventional system is higher than generally estimated in the OER community.

Figure 12 b) shows iR-drop corrected UI-curves normalized to SHE potential, measured in SFC, as comparison. As well as in the conventional system one sees the shift towards lower potentials.

Supplementary Note 9. Corrosion processes in MEA due to lowered pH.

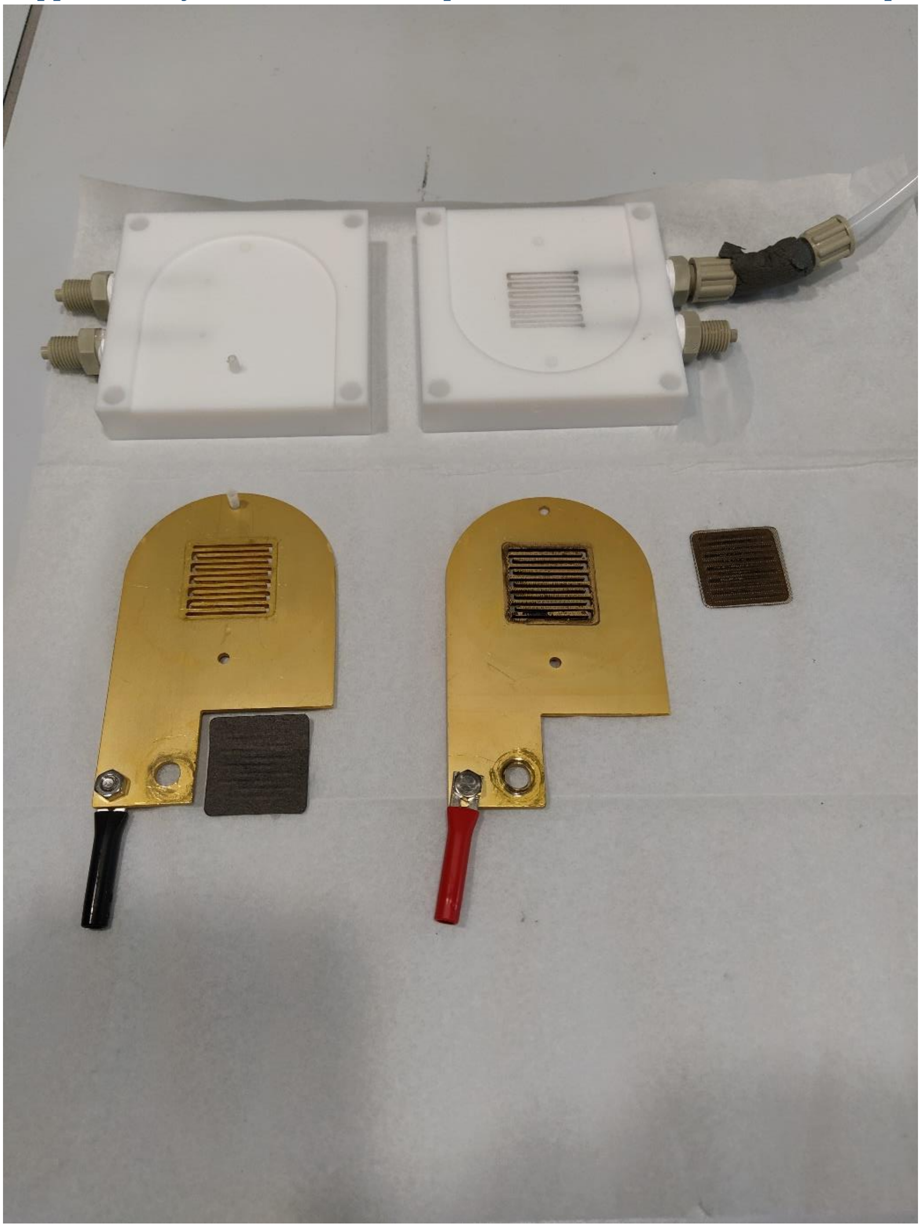

Figure 3 Flow fields and current collectors after 48 h of constant current measurement at 2 A cm^{-2}. The flow field from the cathode side (left) is virtually unchanged while flow field and porous transport layer from the anode (right) side are heavily corroded.

While the precipitation-free MEA cell setup is resistant to normal operation for several days to weeks without noticeable change, damages at the flow fields and current collectors are visible after 48h of continuous operation in 0.1 M H_2SO_4. The setup after the 48h test is shown in Figure 3. While the cathode side is visibly unaltered, corrosion damage at the anode side is clear.

References:

- 1. Shkirskiy, V., et al., *On the Time Resolution of Electrochemical Scanning Flow Cell Coupled to Downstream Analysis.* Journal of The Electrochemical Society, 2019. **166**(16): p. H866-H870.
